# Medical management of acute partial skin necrosis following nipple-sparing mastectomy using an M101-based oxygenating dressing: Two case reports

**DOI:** 10.1016/j.jpra.2025.12.008

**Published:** 2025-12-13

**Authors:** Martin Lhuaire, Victor Pozzo, Enrica Bentivegna, Henri Azais, Tamer Awan, Ignacio Garrido, Laurent Lantieri

**Affiliations:** a Department of Plastic, Reconstructive and Aesthetic Surgery, Hôpital Européen Georges Pompidou, APHP, Université Paris Cité, Paris, France; b Paris Cardiovascular Research Center – Inserm U970, In-vivo Imaging Research Lab, Hôpital Européen Georges Pompidou, Université Paris Cité, Paris, France; c Department of Anatomy and Organogenesis, School of Medicine, Université Paris Cité, Paris, France; d Department of Oncological Gynaecological and Breast Surgery, Hôpital Européen Georges Pompidou, APHP, Université Paris Cité, Paris, France

**Keywords:** Nipple sparing mastectomy, Autologous microsurgical breast reconstruction, Skin necrosis, Oxygenating dressing (M101), Hemhealing®

## Abstract

Postmastectomy skin-flap necrosis after nipple-sparing mastectomy (NSM) is common and exceeds rates after skin-sparing mastectomy (SSM) (0–19.5 %), influenced by patient factors and modifiable intraoperative variables. Presentations range from superficial necrosis to full-thickness necrosis requiring surgical debridement. These events drive reoperation or implant loss, may delay adjuvant therapy, and burden patients psychologically. Clinical examination and ancillary tests poorly discriminate depth; thus, management usually entails close observation until demarcation, followed by targeted intervention. M101, a hemoglobin-based oxygen carrier, demonstrates anti–ischemia–reperfusion effects and promise in advanced wounds. We report two NSM cases with partial skin-flap and nipple–areola complex necrosis managed with an M101-based oxygenating dressing (HemHealing®; Hemarina SA, Morlaix, France), with ultimately no surgical debridement required.

## Cases presentation

Case 1. A 47-year-old woman, smoker (10 cigarettes/day), with undifferentiated nasopharyngeal tumor treated in 2022 and prior right breast invasive squamous cell carcinoma treated in 2016 and 2023 with radiotherapy, presented with bilateral disease, high-risk clinical features, and negative oncogenetics. She underwent bilateral NSM through latero-thoracic incisions using scissor dissection after tumescent adrenaline infiltration,^1^ with immediate bilateral PAP-flap reconstruction anastomosed to the circumflex scapular vessels because abdominal laxity precluded DIEP. On postoperative day (POD) 2, ischemia of both postmastectomy skin flaps and nipple-areola complexes (NACs) was noted. Therapy with the M101 oxygenating dressing was initiated (After Agence Nationale de Sécurité du Médicament approval; AAC 1070697), applied in a thick layer and covered by a secondary non-occlusive dressing (Mepore®, Mölnlycke Health Care AB, Gothenburg, Sweden) every 48 h after gentle cleaning (Supplemental Figure 1). The skin progressively improved, no operative debridement was required, and applications continued until POD17. Complete healing occurred by POD21, with mild residual dyschromia at 3 months (Supplemental Figure 2).

**Fig. 1:**
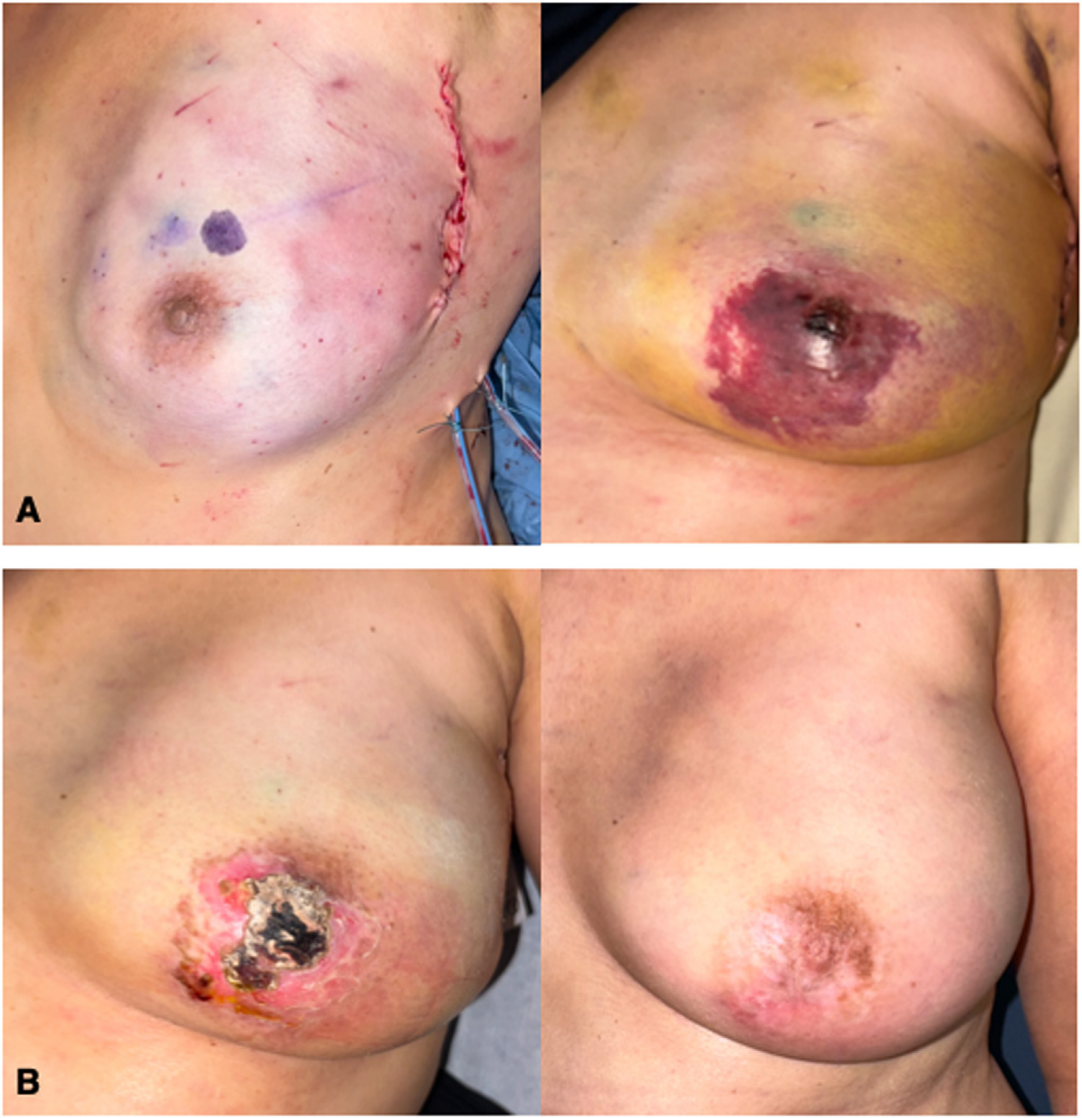
Immediate postoperative appearance of the nipple areola complex and the postmastectomy skin flap, left breast. Postoperative day 6 (A). Postoperative day 19, note the appearance of the skin necrosis. Seven-months postoperative outcome (B).

Case 2. A 55-year-old non-smoking woman with bifocal invasive ductal carcinoma underwent left NSM as previously described,^1^ with immediate DIEP reconstruction anastomosed to the circumflex scapular vessels. On POD1, ischemia of the skin flaps and NAC was observed. M101 treatment began on POD2 (ANSM approval; AAC 1105202), with daily applications following the same protocol (Supplemental Figure 1). By day 19, superficial necrosis warranted minor office debridement, revealing a 1-cm full-thickness focus. No surgical revision was necessary. Complete healing was achieved by day 76, with favorable cosmetic outcomes at 7-month follow-up (Fig. 1).

## Discussion

Although NSM offers superior aesthetics, it carries higher ischemic risk than skin-sparing mastectomy, particularly at the NAC, leading to early reoperation, infection, delayed healing, and compromised outcomes.^2^, ^3^ In our two cases, early use of an M101-based oxygenating dressing was associated with arrest of progression to full-thickness necrosis and avoidance of operative debridement. Patient 1 had active smoking, low body mass index, prior radiotherapy, and scarred areolae; Patient 2 had none. Despite preventive measures, both developed early postoperative ischemia. Favorable courses followed with applications every 24 or 48 h of M101 oxygenating dressing; neither required revision surgery. Adequate oxygenation underpins aerobic metabolism, mitigates ischemia–reperfusion injury, and supports repair in ischemic wounds. M101, an extracellular oxygen carrier from the marine lugworm *Arenicola marina*, provides oxygen down a gradient without cofactors and has anti-inflammatory, antibacterial, and antioxidant activity.^4^ The M101 oxygenating dressing is presented as a hydrogel incorporating M101 with hyaluronic acid and xanthan gum.^5^ Treatment was simple, well tolerated, and followed by complete healing on postoperative days 21 and 76 after 17 and 21 days of use, respectively, without surgical debridement. Hyperbaric oxygen therapy (HBOT) was not used owing to lack of local availability; in that context our objective was to document feasibility and local wound evolution with an M101 hydrogel. No other topical agents (petroleum emollients, honey, collagenase, heparin, or silver sulfadiazine) were applied. Secondary dressings were non-occlusive adhesive dressings. This very small, uncontrolled series limits generalizability; spontaneous resolution of superficial necrosis remains possible, and outcomes cannot be ascribed solely to the intervention. Objective perfusion monitoring was not used (e.g., indocyanine green angiography or transcutaneous oxygen), hindering precise assessment; future studies should integrate such tools to refine indications and timing. These observations occurred in immediate autologous reconstruction (DIEP or PAP) beneath the mastectomy skin envelope, where indirect perfusion from the underlying flap to the NAC may occur. Cost-effectiveness and access require evaluation before routine adoption, findings may not extrapolate to implant-based reconstruction, and long-term effects on pigmentation, scarring, and NAC sensitivity remain to be defined.

## Conclusion

Overall, M101 oxygenating dressing use coincided with improved local oxygenation, nonprogression to full-thickness necrosis, and avoidance of repeat surgery; controlled multi-center evaluation is warranted.

## Role of funding source

None.

## Ethical approval and consent

This report was conducted in accordance with the Declaration of Helsinki. Institutional approval was obtained by Agence Nationale de Sécurité du Médicament – ANSM; AAC number 1070697 and 1105202). Written informed consent for publication of clinical data and images was obtained from both patients.

## Authors contributions

All persons listed as authors have contributed substantially to the design, performance, analysis, and reporting of this work.

ML, VP, EB, HA, TA, IG, LL: designed study, collected data, analyzed data, wrote paper.

## Declaration of competing interest

LL has received a speaker honorarium from the Hemarina SA, Morlaix, France. All other co-authors have no conflict of interest to disclosed.

## Appendix Supplementary materials

Image, application 1

Image, application 2
