# Supplementary material for: Medical management of acute partial skin necrosis following nipple-sparing mastectomy using an M101-based oxygenating dressing: Two case reports
Source: JPRAS Open. 2025 Dec 13;48:434–7. doi: 10.1016/j.jpra.2025.12.008 (PMC12803877; doi:10.1016/j.jpra.2025.12.008)
Supplement: Supplementary file 1 [file mmc1.docx]

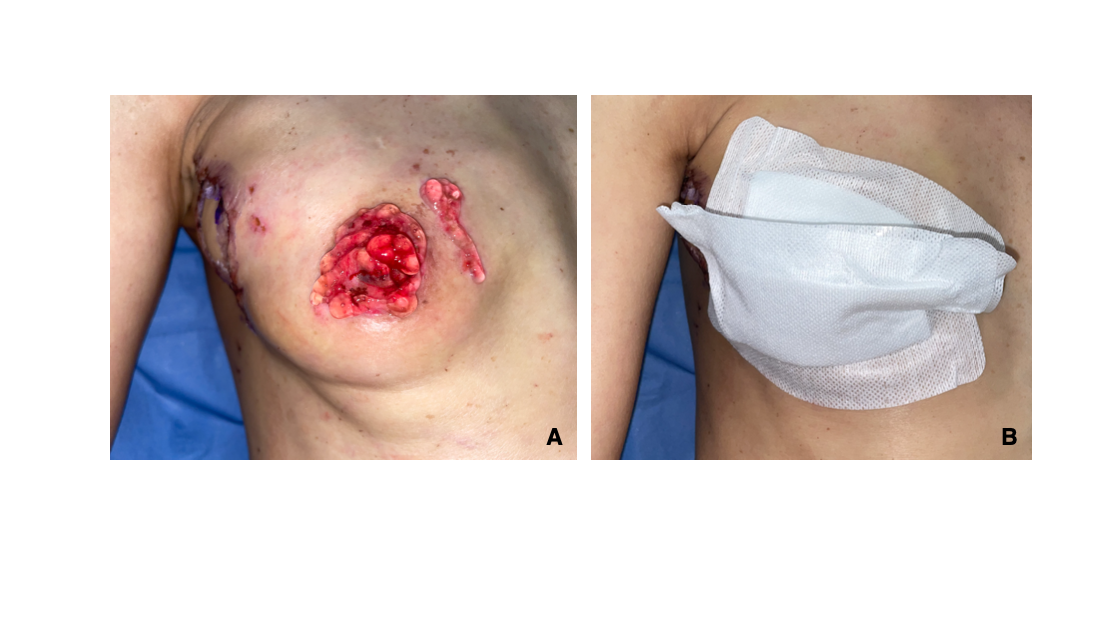


**Supplemental Figure 1 legend:** Application of the M101 hydrogel on the patient 1 right breast on postoperative day 11 after cleaning by saline serum and soap with no mechanical cleansing (day 9 post-treatment by M101 hydrogel) A. Application of the secondary non-occlusive dressing (Mepore® Mölnlycke Health Care AB, Gothenburg, Sweden) B.
