# Supplementary material for: Medical management of acute partial skin necrosis following nipple-sparing mastectomy using an M101-based oxygenating dressing: Two case reports
Source: JPRAS Open. 2025 Dec 13;48:434–7. doi: 10.1016/j.jpra.2025.12.008 (PMC12803877; doi:10.1016/j.jpra.2025.12.008)
Supplement: Supplementary file 2 [file mmc2.docx]

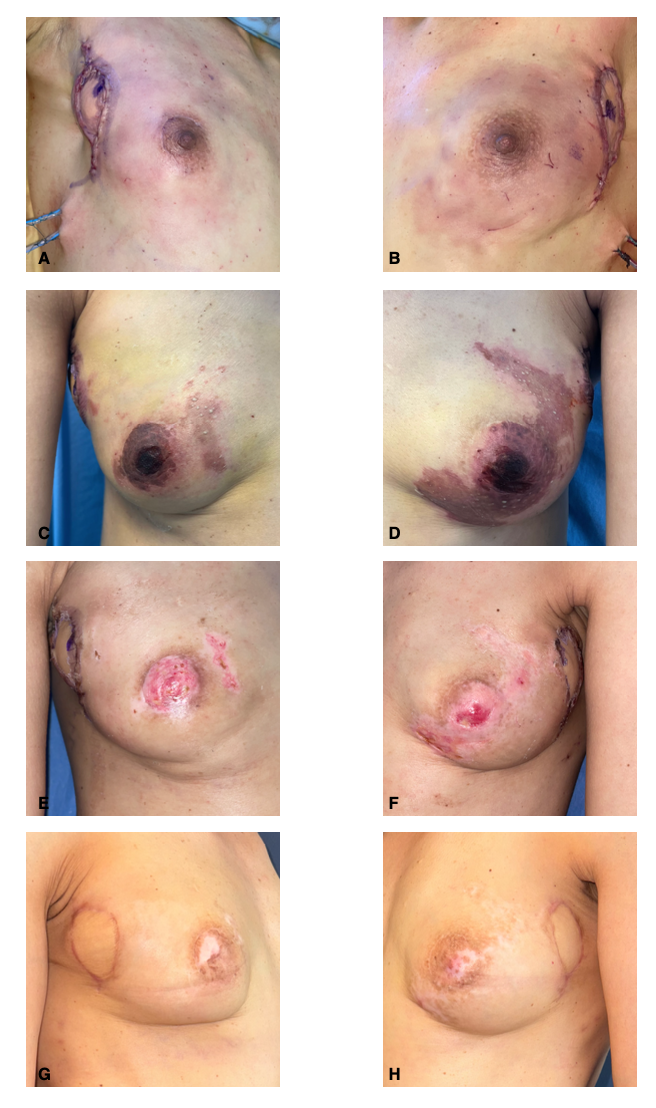


**Supplemental Figure 2 legend:** Immediate postoperative appearance of the nipple areola complex and the postmastectomy skin flap, left breast. Postoperative day 6 **(A)**. Postoperative day 19, note the appearance of the skin necrosis. Seven-months postoperative outcome **(B).**
